# Elixir Iodo-Bromide of Calcium Compound in Scrofulous Induration of the Nose

**Published:** 1873-07

**Authors:** X. T. Bates

**Affiliations:** New Lebanon, N. Y.


ARTICLE II.—*Elixir Iodo-Bromide of Calcium Compound in Scrofulous Induration of the Nose.* By X. T. BATES, M.D., New Lebanon, N. Y.

Was called in February, 1872, to visit Mrs. R. Found her suffering from a troublesome tumor in the right naris, of some twelve months standing, so large as to disfigure the face, accompanied with intolerable burning and itching sensations, which had obstinately resisted every method of medication to which she had been subjected, which I learned comprised both discutient topics and alteratives, and anti-scrofulous remedies. I suggested the use of several lotions and internal medicines, no one of which she appeared inclined to favor, remarking, "it is almost needless for me to use that which has already proven worthless in my case—give me a new prescription." I then prescribed as follows:

R. Elix. Iodo-Bromide Calc. Co., - - - - - o j.

Sig. Take one teaspoonful one hour before each meal; after one week increase the dose to two teaspoonfuls.

This was sufficient to effect a cure which I will pronounce permanent, inasmuch as there has been no return of her difficulty since its disappearance nearly a year since.

I have no hesitation in pronouncing the elixir iodo-bromide of calcium comp., introduced to the profession by Tilden & Co., the most efficient and satisfactory anti-scrofulous preparation I have ever used, but the sphere of its usefulness is by no means confined to scrofula. Aside from this special usefulness it has an application as diversified as the term *alterative* can make it.

